# Gene expression changes throughout the life cycle allow a bacterial plant pathogen to persist in diverse environmental habitats

**DOI:** 10.1371/journal.ppat.1011888

**Published:** 2023-12-19

**Authors:** Roger de Pedro-Jové, Jordi Corral, Mercedes Rocafort, Marina Puigvert, Fàtima Latif Azam, Agustina Vandecaveye, Alberto P. Macho, Carlos Balsalobre, Núria S. Coll, Elena Orellano, Marc Valls

**Affiliations:** 1 Department of Genetics, Microbiology and Statistics, University of Barcelona, Barcelona, Catalonia, Spain; 2 Centre for Research in Agricultural Genomics (CSIC-IRTA-UAB-UB), Bellaterra, Catalonia, Spain; 3 Área Biología Molecular, Facultad de Ciencias Bioquímicas y Farmacéuticas, Universidad Nacional de Rosario and Instituto de Biología Molecular y Celular de Rosario, Consejo Nacional de Investigaciones Científicas y Técnicas (IBR-UNR-CONICET), Rosario, Santa Fe, Argentina; 4 Shanghai Centre for Plant Stress Biology, CAS Centre for Excellence in Molecular Plant Sciences, Chinese Academy of Sciences, Shanghai, China; The Ohio State University, UNITED STATES

## Abstract

Bacterial pathogens exhibit a remarkable ability to persist and thrive in diverse ecological niches. Understanding the mechanisms enabling their transition between habitats is crucial to control dissemination and potential disease outbreaks. Here, we use *Ralstonia solanacearum*, the causing agent of the bacterial wilt disease, as a model to investigate pathogen adaptation to water and soil, two environments that act as bacterial reservoirs, and compare this information with gene expression *in planta*. Gene expression in water resembled that observed during late xylem colonization, with an intriguing induction of the type 3 secretion system (T3SS). Alkaline pH and nutrient scarcity—conditions also encountered during late infection stages–were identified as the triggers for this T3SS induction. In the soil environment, *R*. *solanacearum* upregulated stress-responses and genes for the use of alternate carbon sources, such as phenylacetate catabolism and the glyoxylate cycle, and downregulated virulence-associated genes. We proved through gain- and loss-of-function experiments that genes associated with the oxidative stress response, such as the regulator OxyR and the catalase KatG, are key for bacterial survival in soil, as their deletion cause a decrease in culturability associated with a premature induction of the viable but non culturable state (VBNC). This work identifies essential factors necessary for *R*. *solanacearum* to complete its life cycle and is the first comprehensive gene expression analysis in all environments occupied by a bacterial plant pathogen, providing valuable insights into its biology and adaptation to unexplored habitats.

## Author summary

Most bacterial pathogens have environmental populations outside their hosts that cause disease outbreaks. Very little is known about the genes necessary for adaptation and survival in these conditions because research on pathogenic bacteria has focused on their interaction with the eukaryotic host. In this work, we investigate global gene expression of the plant pathogen *Ralstonia solanacearum* in the two key environmental habitats that it occupies: water and soil. We describe extensive transcriptional reprogramming in response to each environment and unveil a number of candidate genes for adaptation to these harsh conditions. We discover that a costly virulence determinant (the type III secretion system) is transiently induced when bacteria are incubated in natural waters and identify pH as the main signal triggering this response. We propose that water conditions mimic those encountered in the plant xylem fluid and this enables the bacterium to be primed for infection. We also show for the first time that *R*. *solanacearum* induces in the soil the degradation of aromatic compounds and stress-response genes. We demonstrate that the oxidative stress response is essential for survival in this habitat.

## Introduction

Bacterial pathogens have complex life cycles, often including free-living stages in soil or in water bodies [1–3], where they persist in preparation for eventual transmission to a new susceptible host [3]. Therefore, pathogen success depends on its ability to adapt to diverse environments [4]. For instance, pathogenic bacteria can sense and respond to temperature changes, nutrient availability, pH, and osmolarity among other environmental fluctuations [5,6].

*Ralstonia solanacearum* is a globally dispersed pathogen with a complex lifestyle. This bacterium is often considered as soil-borne because it can persist for long periods of time in this environment [7] interacting with the soil microbiome [8]. *R*. *solanacearum* invades plants through natural wounds in the roots [9], occupies the root intercellular spaces or apoplast [9] and eventually colonises the xylem vessels [10], where massive bacterial multiplication accompanied by exopolysaccharide (EPS) production causes vasculature occlusion and plant wilting [11,12]. At the last stages of infection, the pathogen spreads to other tissues and is finally released back to the soil from dead plant material [10]. Contaminated waterways and irrigation waters are an important route for *R*. *solanacearum* dispersal [13] as it can survive for years in water remaining infective [14,15]. Bacterial wilt disease affects over 200 plant species [16] and leads to significant yield losses in important crops such as potato, tomato, peanut, and banana [17].

As in most gram-negative animal and plant pathogens, the major pathogenicity determinant in *R*. *solanacearum* is the type 3 secretion system (T3SS)[18–21]. This system injects bacterial proteins called type 3 effectors (T3E) directly into the eukaryotic host cells to hijack the cellular machinery for bacterial benefit [22]. In *R*. *solanacearum*, expression of the *hrp* genes -encoding the T3SS- is controlled by a membrane receptor (PrhA), a signal transducer (PrhI) and the transcriptional regulators PrhJ, HrpG, and HrpB [23]. HrpB integrates the plant contact induction and other metabolic cues, and directly activates transcription of the T3SS genes [24]. In contrast to other bacterial plant pathogens where expression of the T3SS genes seems to be restricted to early events in the infection process [25–27], in *R*. *solanacearum* this system and its associated effectors is transcribed throughout plant infection [28,29]. Other factors that contribute to *R*. *solanacearum* virulence are flagella and type IV pili mediated motility, cell wall degrading enzymes, reactive oxygen species (ROS)-detoxifying enzymes, and exopolysaccharide (EPS)[30].

Global gene expression studies have provided invaluable information on the components required for bacterial adaptation to ecological niches inside and outside of their hosts [31]. Animal bacterial pathogens such as *Streptococcus pneumoniae*, *Listeria monocytogenes* or *Legionella pneumophila* have been shown to finely tune their genetic programmes to endure the conditions found in the air, soil or waterways [31–33]. In the case of bacterial plant pathogens, gene expression studies have been mostly restricted to *in vitro* conditions [24,34,35] or precise infection stages [26,36,37].

We recently carried out the first study that explores gene expression in a plant bacterial pathogen inside the host plant in different compartments and at different disease stages [28]. The only study comparing transcriptomic profiling of a bacterial plant pathogen inside and outside their host was performed in *Pseudomonas syringae* comparing the epiphytic and apoplastic life stages [38]. Bacteria on the leaf surface showed an upregulation of motility genes and chemosensing among other genes to counterattack plant defences and prepare for plant infection [38].

Gene expression in environmental niches or throughout the whole bacterial life cycle has not been described for any bacterial plant pathogen. In this study, we have analysed *R*. *solanacearum* UY031 global gene expression in environmental conditions outside of the plant to identify the main biological processes governing adaptation of this pathogen to soil and water. We hypothesised that virulence-associated genes would not play a significant role in the adaptation and persistence of the bacterium in these environments and instead, genes required for the metabolism of alternative carbon sources might be important for survival in these oligotrophic conditions. Our results show an intriguing up-regulation of the T3SS genes in water as well as global changes in bacterial metabolism and nutrient acquisition that take place for the bacterium to survive in soil.

## Results

### *R*. *solanacearum* expression profile in water mimics xylem expression while a unique transcriptional reprogramming is observed in soil

**Fig 1:**
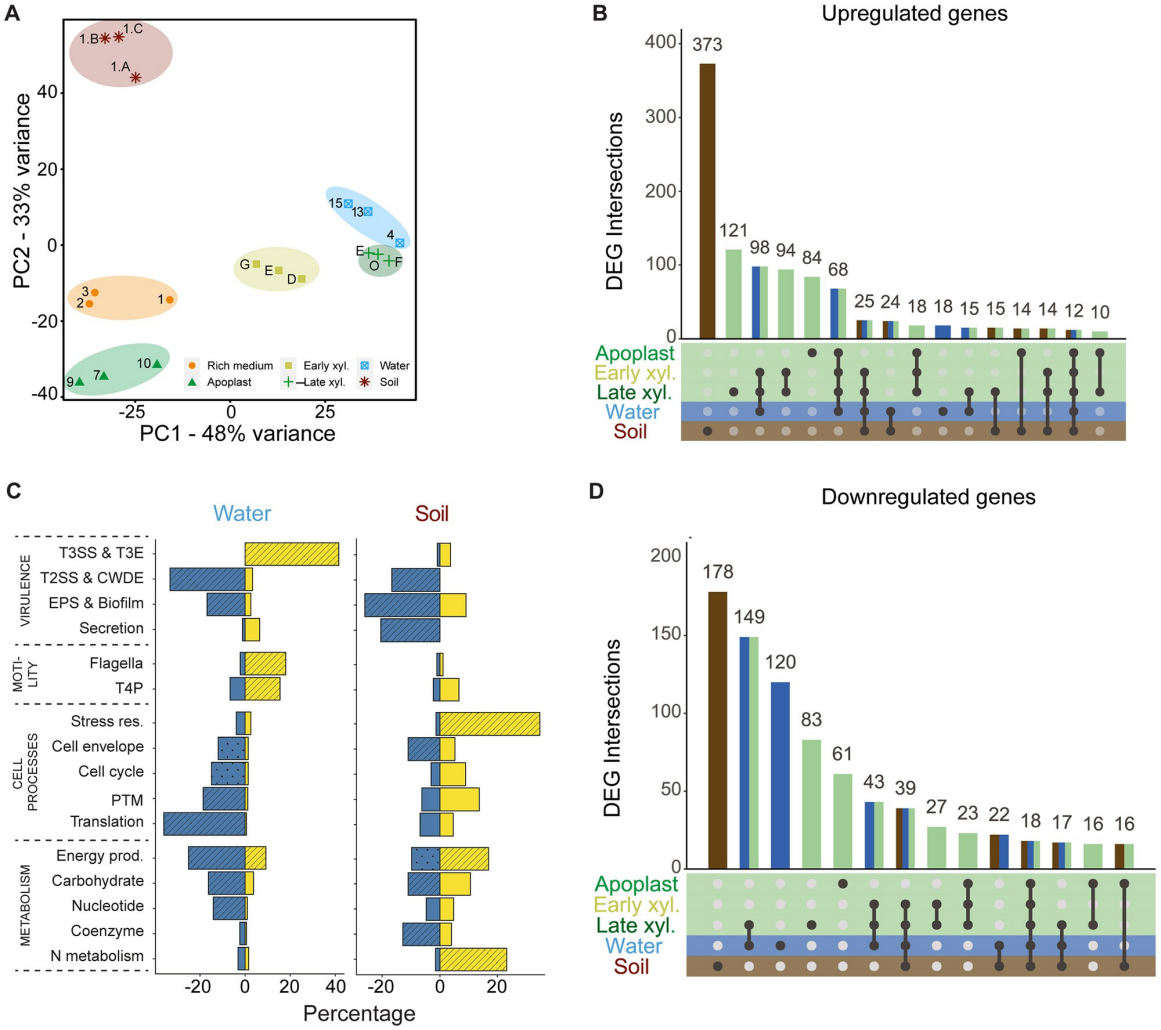
*R*. *solanacearum* transcriptomic profile in *in vivo* conditions. **A)** Two-dimensional Principal Component Analysis representation of expression data for all samples used in this study. Three biological replicates were analysed per condition. **B)** Shared and unique differentially expressed genes (DEG) across two environmental (soil—brown and water—blue) and three in planta conditions (Apoplast, Early and Late—green). Vertical bars represent DEG unique or shared between the indicated conditions (number above each bar). Only interactions with more than 10 genes are shown. DEGs were identified with DEseq2 (p-adj > 0.01, log2 FC ± 1.5) and plotted using UpsetR. **C)** Percentage of up- (yellow) and downregulated (blue) genes for each functional group in water and soil conditions. Categories were generated based on KEGG, COG and Uniprot information and grouped by functional similarity. Only significantly overrepresented categories (hypergeometric test p.value < 0.05 -line pattern- and < 0.01 -dotted pattern) in at least one of the conditions are shown. Short names for categories are as follows: Secretion (Intratraficking and secretion), T4P (Type IV pili), PTM (Post-translational modification), Stress res. (Stress response), Translation (Translation and ribosome), Energy prod. (Energy production).

To investigate the transcriptomic landscape of *R*. *solanacearum* outside plant hosts we chose the environmental conditions where it has been most often isolated: waterways and soil. To investigate expression in water, bacterial cells were resuspended in a spring water (Water A–S1 Table) and recovered after six hours, which was observed to be the most informative time point in previous studies with other bacterial pathogens [31]. For soil samples, *R*. *solanacearum* was inoculated in a natural soil (S2 Table) and total RNA was directly isolated three days later, which is the time point when plant infection occurs in our experimental conditions [28] (S1A Fig). The timepoints used in this study were selected to investigate the transcriptomic response of *R*. *solanacearum* adaptation to a specific environment. To obtain a full gene expression landscape, RNA sequencing reads were analysed together with those previously obtained from *R*. *solanacearum* grown in rich B medium or extracted from infected potato plants at three representative disease stages: growth in the apoplast, in the xylem at the onset of symptoms and in the xylem at late stages when plants were dead [28] (S1A Fig). The principal component analysis (PCA) revealed a robust clustering of all biological replicates and a clear distinction of soil samples from the other conditions (Fig 1A). In contrast, water samples were observed to share similarity with late xylem samples (Fig 1A). Comparison with *in planta* conditions [28] confirmed that gene expression in soil was the most distinct, with 373 genes uniquely upregulated and 178 uniquely downregulated (*p* value of 0.01 and log2-fold change of ≤1.5) in this condition (Fig 1B and 1D and S1 Dataset). On the contrary, 50% of the differentially expressed genes (DEGs) in water were shared with at least one of the *in planta* conditions (Fig 1B and 1D and S3 Table).

### The type 3 secretion system is strongly induced in water through a novel signalling pathway

Genes upregulated in water compared with the reference condition rich B medium were enriched with the functional category “T3SS” (> 40%) followed by “Flagella and Type IV pili” (~20%) (Fig 1C and S4 Table). In contrast, downregulated genes were enriched in other functional categories associated with virulence such as “Type 2 secretion system (T2SS) and Cell wall degrading enzymes” (~ 33%) as well as “EPS and biofilm” (~20%) (Fig 1C). Moreover, enrichment of downregulated genes within the categories “Translational and ribosome” (36%) and “Energy production” (25%) suggest a metabolic shut-down (Fig 1C and S4 Table). Alternative classification of genes based in gene ontology (GO) terms and KEGG pathways yielded similar category enrichment results (S2 Fig and S5 Table).

**Fig 2:**
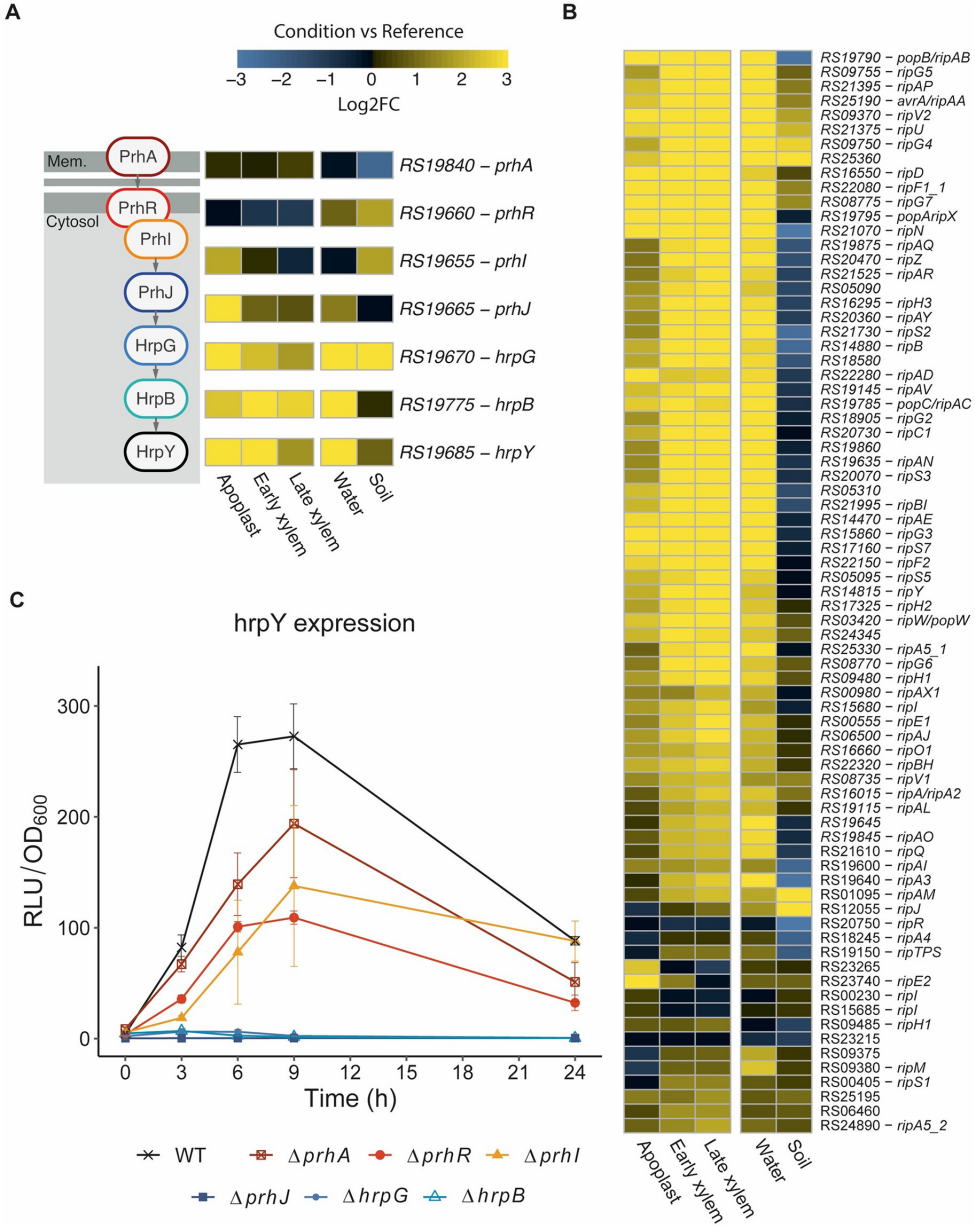
Induction of type 3 secretion system (T3SS) genes in water. **A**) Representation of the main components of the T3SS regulatory cascade and their expression in different conditions (log2 fold change with respect to rich B medium). **B**) Heatmap representation of the log2 fold change with respect to growth in rich B medium for type 3 effector genes in all conditions. The colour palette ranges from blue (downregulated) to yellow (upregulated genes) as indicated in the key. Locus names are presented without the preceding letters RSUY_. **C**) Time-course expression of the *PhrpY*:*Lux* reporter in strains disrupted for the different T3SS regulatory genes after resuspension in water. *R*. *solanacearum* cultures grown overnight in rich B medium were washed and diluted to OD_600_ = 0.1 in water and luminescence and OD_600_ values were measured over a 24 h period. Relative luminescence units (RLU) were normalised by bacterial concentration (OD_600_). Original RLU values were divided by 1000.

T3SS genes are essential for *R*. *solanacearum* virulence and are strongly induced inside the plant [18,28,29]. It was thus surprising that *hrpG* and *hrpB*, the central activators of the T3SS regulatory cascade as well as most type 3 structural components (including *hrpY* gene which encodes the main T3SS pilus component) (Fig 2A) and most type 3 effector genes appeared similarly upregulated in bacteria incubated in water (Fig 2B). To precisely elucidate the induced signalling pathway, we measured expression of *hrpY* in different strains disrupted for each of the known components and regulators of the T3SS regulatory cascade. Luminescence quantification of the *PhrpY*:*lux* reporter strain showed that induction in water peaked after 6 to 9 hours and was very low after 24h. This induction was abolished in the Δ*prhJ*, Δ*hrpG* or Δ*hrpB* deletion mutants (Fig 2C). Thus, the newly discovered water induction signal is integrated in the T3SS regulatory cascade at the level of the PrhJ transcriptional regulator. The same result was obtained when *hrpB* induction was measured in different mutant backgrounds (S3 Fig).

### Alkaline pH and starvation are the environmental signals triggering T3SS induction in water

**Fig 3:**
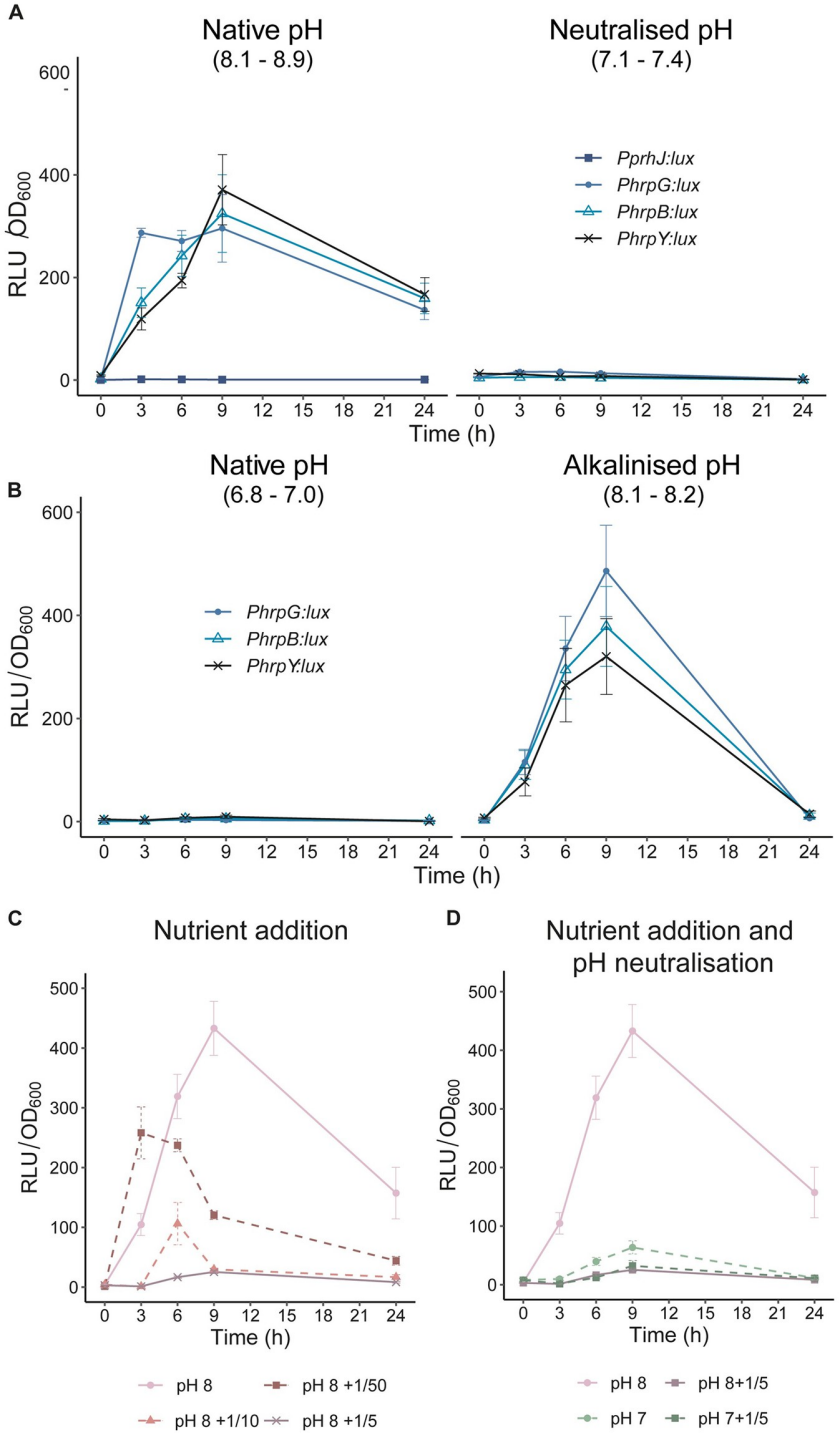
Alkaline pH and starvation induce the type 3 secretion system genes (T3SS). Time-course expression of *R*. *solanacearum* reporter strains in **A**) Native alkaline pH mineral water or the same water adjusted to neutral pH and **B**) Native neutral water or the same water alkalinized to alkaline pH. For each time point, luminescence is indicated in relative units divided by 1000 (RLU) normalised by OD_600_. **C**) Time-course expression of *hrpB* in water or the same water supplemented with increasing concentrations of rich B medium. Numbers indicate the fraction of rich B medium. **D**) Time-course expression of *hrpB* in water, water neutralized to pH = 7, water supplemented with 1/5 volume of rich B medium, or neutralized water supplemented with 1/5 volume of rich B medium.

Next, we decided to determine the precise environmental cues that induced the T3SS genes in water. Since water is almost depleted of nutrients and the mineral water used for transcriptomic experiments was slightly alkaline (pH ~ 8), we analysed whether these factors affected T3SS gene expression. First, we measured expression of *hrpY* and the upstream regulators involved in T3SS induction in water (*prhJ*, *hrpG* and *hrpB*) in the native spring water or adjusted to neutral pH (Fig 3A). This experiment showed that *hrpG* and *hrpB* and *hrpY*, but not *prhJ*, transcription was induced in water and that this induction was completely abolished at neutral pH (Fig 3A). To prove that this was a general phenomenon, we repeated the experiment in waters from six different natural sources throughout the Iberian Peninsula, five of which were naturally alkaline (pH 8.1 to 8.8) and one neutral (S1 Table). Clear induction of *hrpG*, *hrpB* and *hrpY* was observed in all alkaline waters collected and neutralisation abolished these inductions (S4 Fig). On the contrary, while gene expression was almost undetectable in the naturally neutral water, its alkalinisation to pH 8 resulted in *hrpG*, *hrpB* and *hrpY* induction, demonstrating causality of alkaline pH in the induction of T3SS in water (Fig 3B and S4 Fig).

Next, given that water is a nutrient poor environment, we investigated the importance of water nutrient scarcity in T3SS induction by measuring *hrpB* expression after addition of different volumes of rich B medium into water at inducing alkaline water pH (Fig 3C). Progressive reduction of T3SS gene expression was observed with increasing concentrations of rich B medium added, showing that nutrient addition abolished T3SS induction in water in a direct dose response (Fig 3C).

Finally, the combined effect of the pH- and nutrient-dependent T3SS induction was tested on *hrpB* expression. This experiment revealed that alkaline pH and nutrient scarcity equally impacted T3SS induction and that the nutrient addition mediated repression was epistatic over alkaline pH, since no induction of *hrpB* expression was detected at alkaline pH when nutrients were added (Fig 3D).

### The xylem sap becomes alkalinised during *R*. *solanacearum* infection

**Fig 4:**
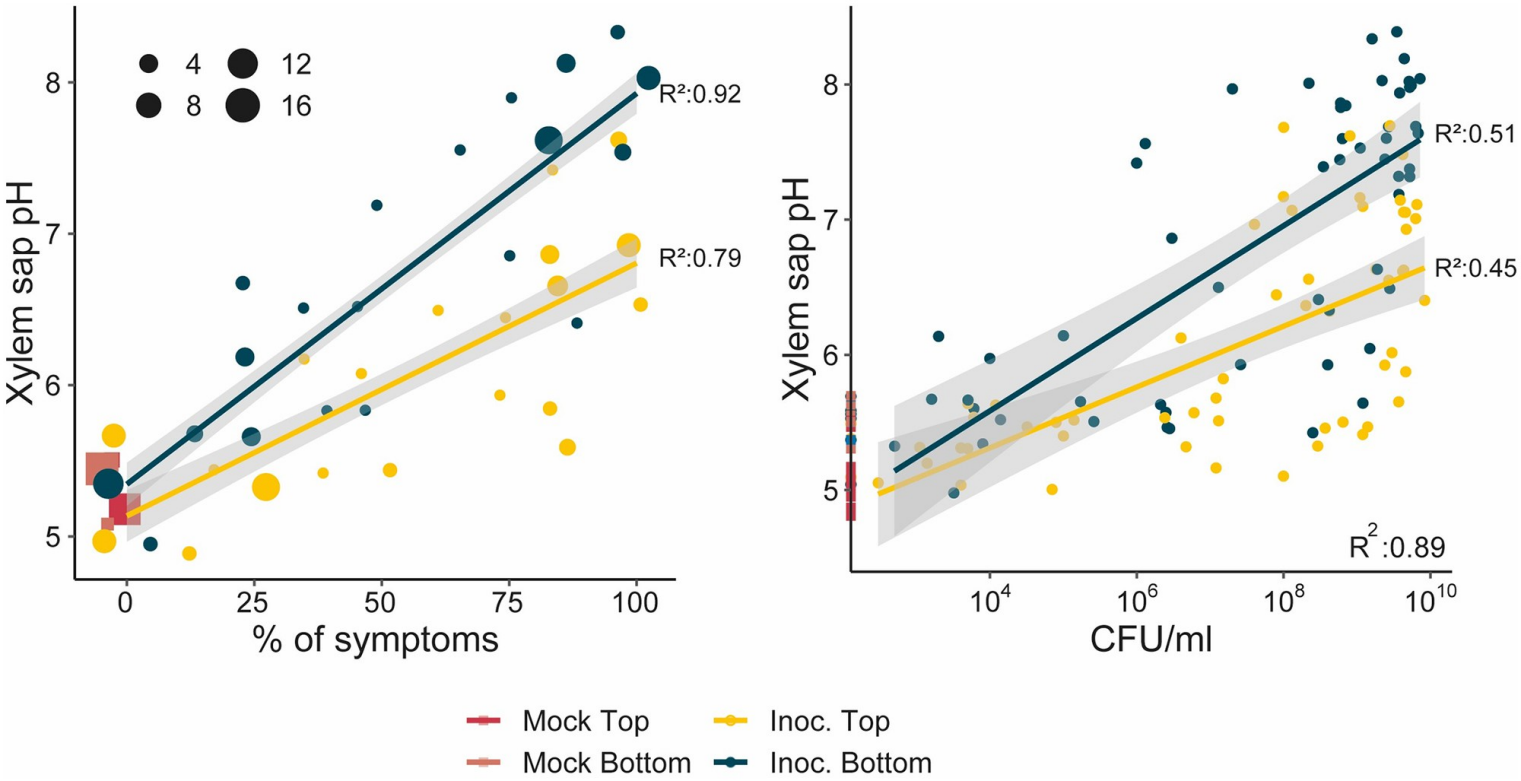
The pH of xylem sap is alkalinised during *R*. *solanacearum* infection. 3-week-old tomato plants were soil drench inoculated with *R*. *solanacearum* or mock treated with water and their symptoms recorded. Xylem samples from mock (square shapes) or inoculated tomato plants (Inoc., round shapes) were collected from the bottom or the top of the stem of plants at different disease stages and xylem pH and bacterial loads measured. Graphs represent xylem pH vs plant symptoms or xylem pH versus *R*. *solanacearum* content in the xylem. Shape sizes is proportional to the number of coincident points. R^2^ scores and lines indicate correlation calculated using linear model (“lm”).

Low nutrients and high pH encountered in water may induce the T3SS because these conditions resemble those encountered by the bacterium *in planta*. It was known that the xylem sap contained relatively limited nutrients [39,40], but the pH found in the xylem during *R*. *solanacearum* infection was unknown. To investigate this, we compared the pH of xylem sap isolated from top or bottom of tomato stems non-inoculated (mock) or inoculated by drenching the soil with a suspension of *R*. *solanacearum* GMI1000. Remarkably, non-inoculated plants showed slightly acidic xylem pH whereas an alkaline pH was observed in almost all diseased plants, reaching pH ~ 8 in heavily wilted or colonised plants. In addition, a positive correlation was observed between xylem pH and both disease symptoms and bacterial loads (Fig 4). In summary, *R*. *solanacearum* infection caused xylem alkalinisation, which was more apparent at the base of the stem than in apical parts, most likely because the bottom of the stem contains higher pathogen loads (Fig 4, 10].

### Stress response genes are strongly induced and support *R*. *solanacearum* adaptation to the soil environment

To understand the dominant biological processes in the soil, *R*. *solanacearum* genes were classified in functional groups, and the percentage of DEGs within each group and their enrichment were calculated (Fig 1C and S4 Table). A major part of genes associated with functional category “Stress response” (35%) and “Nitrogen metabolism” (26%) were upregulated in soil compared with reference condition rich B medium. Interestingly, nitrogen metabolism, which we have previously reported to be upregulated ~ 3 fold during early and late xylem infection [28], was observed to be even more upregulated (~ 5 fold) in the soil environment (S5 Fig). In contrast, most downregulated genes were classified in different functional categories associated with virulence (Fig 1C and S4 Table). Classification of genes based in GO term and KEGG pathways and enrichment analyses yielded similar results (S2 Fig and S5 Table), reinforcing their robustness.

To further investigate the transcriptional reprogramming in soil, we performed a closer scrutiny of genes with a log2 fold expression change ≥2 in soil compared with rich B medium (Table 1). We observed that several antibiotic protection/production genes and toxin/antitoxin genes (TA modules) were upregulated in soil. It has been reported that chromosomal TA modules are enriched in the genome of bacterial pathogens and are involved in biofilm formation, multidrug tolerance, cell formation and growth arrest [41]. On the contrary, downregulated genes were associated with plant virulence, many of them encoding EPS formation, type IV pili and secretion system components. In line with this, the two only upregulated genes in this functional category encode the SinR and PuuR repressors that downregulate the expression of biofilm formation and putrescine biosynthesis, respectively (Table 1).

**Table 1:** A subset of marker genes from soil classified according to their putative function. Locus names are presented without the preceding letters RSUY_. LFC stands for log2 fold change in expression with respect to growth in rich B medium.

Category	Locus_tag	LFC	Name	Protein description
**Stress response**				
Oxidative stress	RS10090	7	*ahpC2*	alkyl hydroperoxide reductase subunit C
	RS17495	4.3	*ahpC*	alkyl hydroperoxide reductase subunit C
	RS17500	4.5	*ahpF*	alkyl hydroperoxide reductase subunit F
	RS04870	6.2	*katG*	catalase/peroxidase HPI
Metal homeostasis	RS00665	4.8	*copA*	heavy metal translocating P-type ATPase
	RS00670	4.6	*hmrR*	Cu(I)-responsive transcriptional regulator
	RS07025	4.0	*scU*	Fe-S cluster assembly scaffold
Other/Putative	RS00120	3.7	*cstA*	carbon starvation protein A
	RS03790	3.3	*dps2*	DNA starvation/stationary phase protection protein
**Soil metabolism**				
Degradation of aromatics	RS02905	3.1	*paaI*	hydroxyphenylacetyl-CoA thioesterase
	RS02910	3.1	*paaG*	2-(1,2-epoxy-1,2-dihydrophenyl)acetyl-CoA isomerase
	RS18560	3.1	*paaB*	1,2-phenylacetyl-CoA epoxidase subunit B
	RS18565	2.7	*paaC*	phenylacetate-CoA oxygenase subunit
Glyoxylate cycle	RS08780	3.0	*icl/aceA*	isocitrate lyase
	RS08800	2.8	*aceB*	malate synthase A
Nitrogen metabolism	RS15890	8.0	*norB*	nitric-oxide reductase large subunit
	RS15900	6.4	*nirK/aniA*	Nitrite reductase
	RS00425	5.6	*hmpX*	Detoxify nitric oxide
	RS17970	5.5	*narH*	Nitrate reductase
	RS17965	5.3	*narG*	Nitrate reductase
Respiration	RS22145	6.7		cytochrome C oxidase subunit IV family protein
	RS08370	5.5	*ccoQ*	Cbb3-type cytochrome oxidase component FixQ
	RS22130	5.4	*coxN/ctaD*	Alternative cytochrome c oxidase subunit 1
	RS08365	5.0	*ccoO*	Cytochrome-c oxidase, cbb3-type subunit II
Other	RS11540	2.7		acyl-CoA dehydrogenase
	RS12470	4.2	*clpA*	ATP-dependent Clp protease
	RS10020	2.4	*clpP*	ATP-dependent Clp protease
	RS22610	7.6		H-NS histone family protein

**Fig 5:**
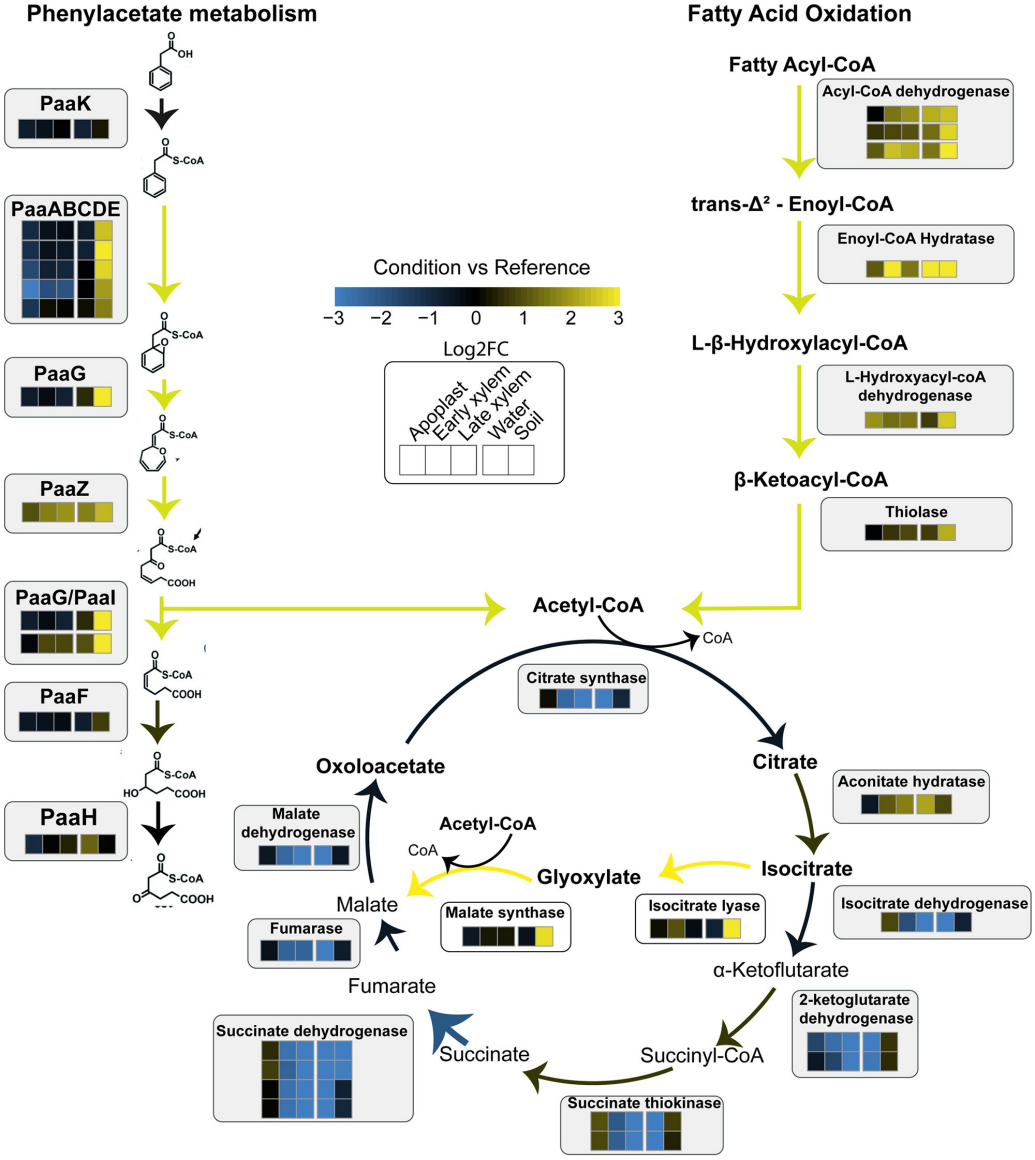
Induction of glyoxylate cycle genes in soil. Representation of phenylacete catabolism pathway and fatty acids oxidation pathway that lead to the production of an acetyl-CoA pool that feeds the glyoxylate cycle. Squares are a heatmap representation of the log2 fold change in expression with respect to growth in rich B medium. The colour palette ranges from blue (downregulated) to yellow (upregulated genes) as indicated in the key. *paak*: RSUY_RS02900; *paaA*: RSUY_RS18555; *paaB*: RSUY_RS18560; *paaC*: RSUY_RS18560; *paaD*: RSUY_RS18570; *paaE*: RSUY_RS1857; *paaG*: RSUY_RS02910; *paaZ*: RSUY_RS11535; *paaG*: RSUY_RS02910; *paaI*: RSUY_RS02905; *paaF*: RSUY_RS02915; *paaH*: RSUY_RS11390; *Acyl-CoA dehydrogenase*: RSUY_RS13310, RSUY_RS11540, RSUY_RS06380; *Enoyl-CoA Hydratase*: RSUY_RS01105; *L-Hydroxyacyl-coA dehydrogenase*; RSUY_RS09555; *Thiolase*: RSUY_RS09565; *Citrate synthase*: RSUY_RS11275; *Aconitate hydratase*: RSUY_RS17195; *Isocitrate dehydrogenase*: RSUY_RS12515; *2-ketoglutarate dehydrogenase*: RSUY_RS08325, RSUY_RS08325; *succinate thiokinase*: RSUY_RS13215, RSUY_RS13220; *succinate dehydrogenase*: RSUY_RS11285, RSUY_RS11290, RSUY_RS11295, RSUY_RS11300; *Fumarase*: RSUY_RS11110; *Malate dehydrogenase*: RSUY_RS11310; *Isocitrate lyase*: RSUY_RS08780; *Malate synthase*: RSUY_RS08800.

Remarkably, many genes exclusively upregulated in soil belonged to the *paa* (phenylacetate catabolism) cluster associated with the degradation of aromatics. Additionally, genes encoding enzymes associated with the oxidation of fatty acids, such as the enzyme Enoyl-CoA hydratase, were also extensively upregulated in soil (Fig 5). The two genes encoding the key enzymes of the glyoxylate cycle (isocitrate lyase and malate synthase) were upregulated exclusively in this soil environment (Table 1 and Fig 5). Furthermore, the glyoxylate carbo-ligase and the glycerate kinase, enzymes involved in transformation of glyoxylate to pyruvate, were also strongly induced in soil.

**Fig 6:**
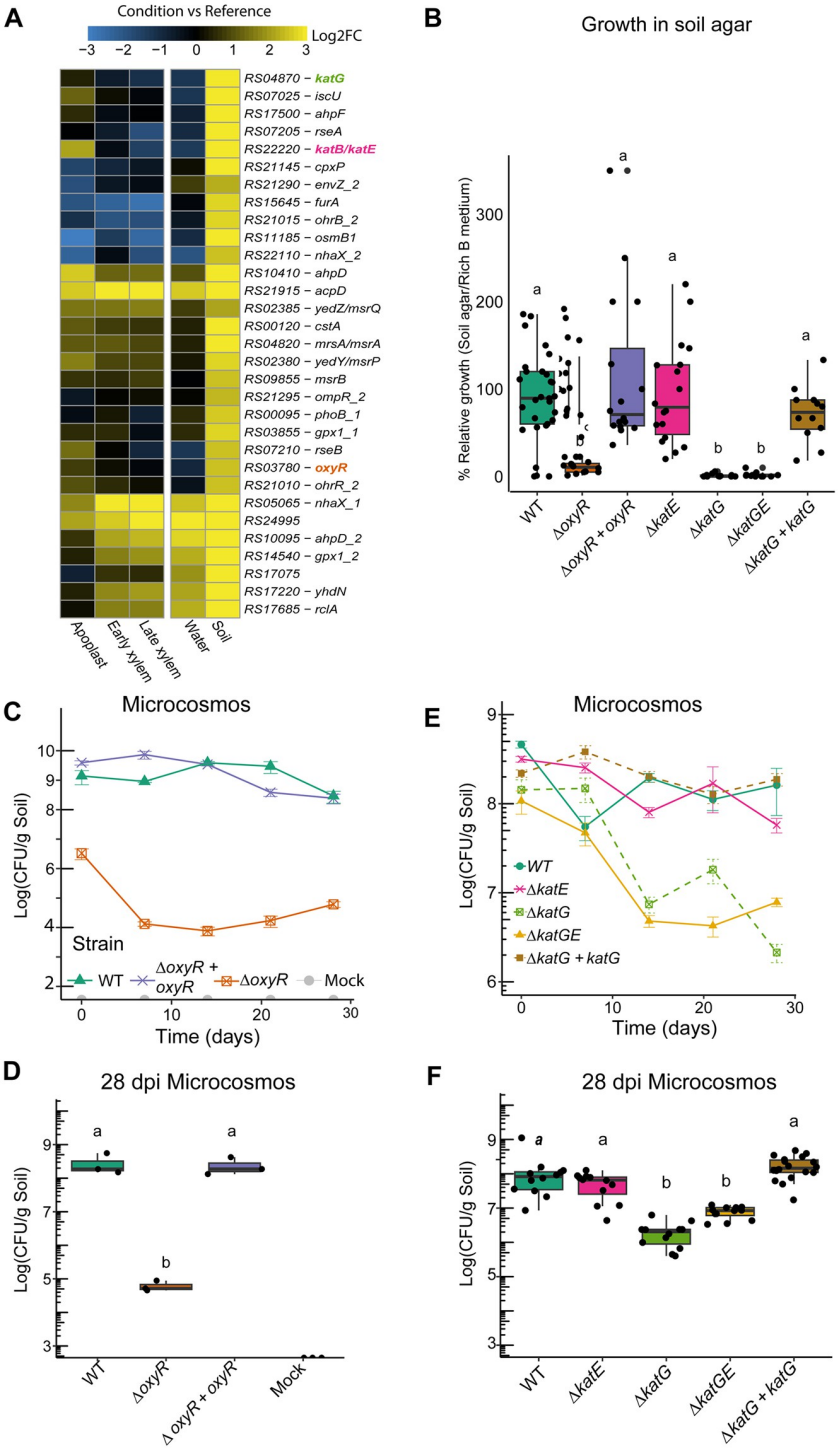
Stress response genes are important for *R*. *solanacearum* adaptation to soil. **A**) Heatmap representation of gene log2 fold change with respect to the rich B medium in the different conditions for stress response genes differentially upregulated in soil. The colour palette ranges from blue (downregulated) to yellow (upregulated genes), as indicated in the key. Locus names are presented without the preceding letters RSUY_. Genes selected for functional characterisation are shown in bold. **B**) Bacterial counts (Log CFU/mL) of wild type *R*. *solanacearum* (WT), oxidative stress regulator (Δ*oxyR*) mutant and single or mutiple catalase mutants (Δ*katE*, Δ*katG*, and Δ*katGE*), and the Δ*oxyR* complemented (Δ*oxyR + oxyR)* and Δ*katG* complemented strains (Δ*katG + katG)* grown in soil agar relative to growth in rich B medium. **C**) Bacterial culturability (Log CFU/mL) in natural soil microcosms of WT, Δ*oxyR* and Δ*oxyR + oxyR*. **D**) Bacterial culturability data (Log CFU/mL) in natural soil microcosms 28 days after inoculation of strains shown in C. **E)** Bacterial culturability (Log CFU/mL) in natural soil microcosms of WT, Δ*katE*, Δ*katG*, and Δ*katGE* and Δ*katG + katG*. Bacterial culturability data (Log CFU/mL) in natural soil microcosms 28 days after inoculation of strains shown in E. Different letters indicate significant differences according to one-way ANOVA (*p-value* < 0.1) followed by TukeyHSD statistical test.

Finally, most genes upregulated in soil were associated with a stress response, which included genes encoding for ROS detoxifying enzymes and genes associated with metal homeostasis (Table 1 and Fig 6A and S6A Fig). To investigate if the expression of genes associated with oxidative stress was exclusive to the soil environment, we performed RT-qPCRs of *R*. *solanacearum* survival in water at a late time point (3 dpi). Results showed that genes involved in ROS detoxification are not induced in water (S7 Fig). To further investigate the importance of stress response related genes for *R*. *solanacearum* fitness in the soil condition, we selected some of the most upregulated genes: *katE* gene encoding a catalase, *katG* encoding a catalase-peroxidase [42] as well as the *oxyR* gene, a global transcriptional regulator controlling many oxidative stress genes [43,44]. We generated gene deletion mutants and plated the different strains on soil-agar plates mimicking soil conditions. Remarkably, *R*. *solanacearum* strains with Δ*oxyR* and Δ*katG* deletions grew significantly less in soil agar than the wild type strain and growth was restored in the complemented strains (Fig 6B). However, no differences were observed between the Δ*katE* strain and the wild type, suggesting no major role of KatE in soil. To further investigate the role of these genes over long-periods of time in soil, the mentioned bacterial strains were directly inoculated in natural soil microcosms at 10^8^ CFUs per g and culturability was measured by plating strains throughout a 28-day period. In line with previous results, strains with Δ*oxyR* and Δ*katG* deletions showed a significant decrease in culturability, a phenotype clearly rescued by complementation, while no effect of the *katE* deletion was observed (Fig 6C, 6D, 6E and 6F). It has been shown that *R*. *solanacearum* can trigger the viable but nonculturable cell state (VBNC) as a survival mechanism in stressful conditions such as growth in soil [45,46]. Therefore, to analyse if the decrease in culturability was due to a loss of viability or bacterial cells remained in a VBNC state, cell viability was assessed by microscopy. Remarkably, no clear viability differences were observed among wild type and *oxyR* and *katG* deletion mutants (S8 Fig) in any microcosms time point, suggesting that most cells remain in VBNC state.

## Discussion

**Fig 7:**
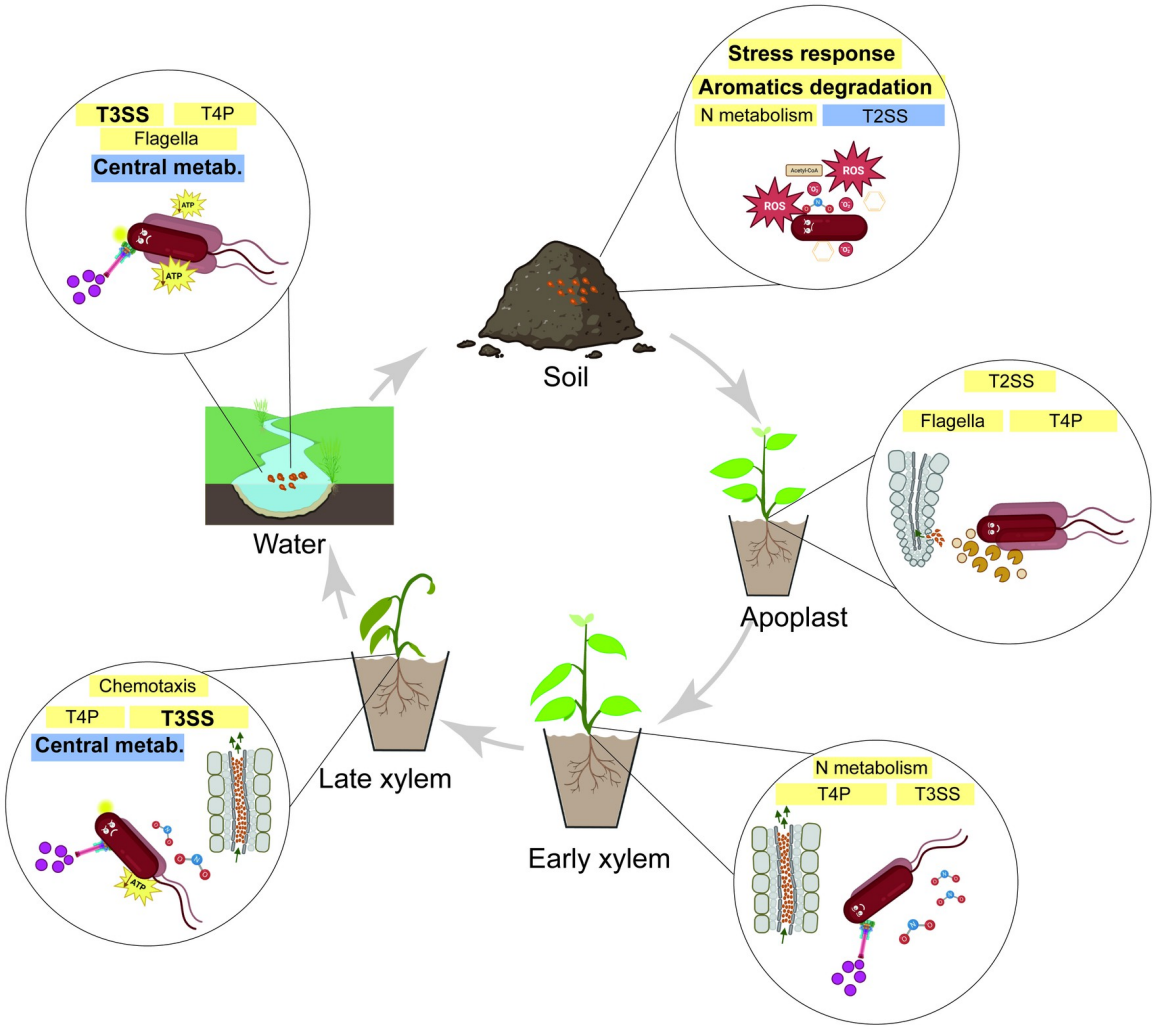
Graphical representation of the main biological processes during *R*. *solanacearum* life cycle. Highlighted processes based on transcriptomic data and enriched categories. N metabolism: Nitrogen metabolism, T2SS: Type 2 secretion system, T4P: Type IV pili, T3SS: Type 3 secretion system, Central metab.: Central metabolism. Drawings created with BioRender.

As an environmental pathogen, *R*. *solanacearum* can spend most of its life outside the plant in soil or waterways, where persistence and survival of the pathogen has been reported [7,15]. The ability of *R*. *solanacearum* to survive in different environments generates a source of inoculum for future disease outbreaks and therefore, research of these unexplored environmental niches is key for disease control [7]. It has been proposed that in this inter-host life the pathogen must cope with many stresses linked to oligotrophic habitats [47]. In this work, we study the changes in gene expression required for successful adaptation to environmental habitats and for parasitic life inside the plant host, shedding light on the biological processes that are key for adaptation and survival throughout its life cycle (Fig 7).

In this study, we have focused on the transcriptomic short-term adaptation in water. Surprisingly, no clear functional categories associated with survival in this environment were identified. Instead, we show that that *R*. *solanacearum* upregulates flagellar activities (Fig 1C and S2 Fig) in this environment. Interestingly, a similar response was observed in *Campylobacter jejuni* and *L*. *pneumophila*, the only bacterial pathogens whose gene expression had been determined in water [31]. *R*. *solanacearum* and *L*. *pneumophila* also share a dramatic repression of genes related to transcription and translation and an induction of virulence factors in these oligotrophic conditions [31] (Fig 1C), suggesting that in water these pathogens enter a quiescent state primed for host infection.

Surprisingly, the *R*. *solanacearum* transcriptional profile in water is very reminiscent of that observed *in planta* at late infection stages (Fig 1A, 1B and 1D). The main commonalities are an overall metabolic shutdown and an unanticipated induction of T3SS. The severe downregulation of genes involved in protein synthesis (genes encoding both ribosomal proteins and tRNAs), central metabolism and energy production indicates a clear drop in the metabolic activity of the bacterial cells, consistent with the scarce concentration of nutrients present in water. The reported low nutrient concentration in xylem sap of infected cells [48] may explain the similarities found in the transcriptional expression profiles between water and xylem. However, the energy costly induction of the T3SS in water is unexpected. One possible explanation for this is that T3SS induction in water provides a biological advantage to the pathogen. For example, T3SS induction in water could prepare the pathogen for infection of reservoir hosts usually present in riverbanks, such as *Solanum dulcamara*. Another possible explanation is that environmental cues inducing the T3SS system are by chance present in water. We favour the second hypothesis because T3SS induction is transient—almost undetectable after 24 h- and because high pH is the main inducing signal and correlates perfectly with T3SS induction levels throughout xylem infection (Fig 4). Finally, in line with previous research that revealed that T3SS is not expressed in rich B medium [18,49], we observed that T3SS repression upon nutrient addition was epistatic to induction at alkaline pH (Fig 3), indicating that nutrient scarcity is a pre-requisite for T3SS induction.

Our gene expression data clearly show an induction of the T3SS genes and the vast majority of the associated effectors in all *in planta* conditions but especially in the xylem (S6B Fig). This expression pattern clearly suggests the T3SS is not only required during early infection. Instead, it indicates that the T3SS might be important throughout infection to inject bacterial proteins into the plant host cells -parenchyma or xylem companion cells- to promote virulence during late infection stages [28]. Thus, when *R*. *solanacearum* enters a water course it encounters conditions that mimic those found in the xylem at advanced wilting symptoms, which trigger transient induction of the T3SS genes.

Expression of the T3SS genes is tightly controlled through a cascade of transcriptional regulators induced upon contact with plant cells [23]. Here, we show that a new signal in response to water is integrated in the cascade through the *prhJ* (Fig 2C), most likely post-transcriptionally because *prhJ* transcription is not induced by water (Fig 2A). PrhI and PrhR also seem to influence or be partially required for T3SS expression in water (Fig 2C), illustrating the complexity of the regulatory network.

*R*. *solanacearum* transcriptome response in soil was the most distinct from all conditions in its life cycle. A general shutdown of bacterial metabolism was observed (Fig 1C), reflecting that the soil is an oligotrophic environment for *R*. *solanacearum*. Repression of many genes involved in ribosome formation and protein synthesis and activation of genes coding for *lon* and *clp* proteases indicate amino acid starvation in soil [50]. In addition, *dps*, known to be induced upon starvation in *R*. *solanacearum* [51] and crucial for protection under challenging conditions in different bacteria [52,53] was highly upregulated.

*R*. *solanacearum* displayed a specific metabolic reprogramming to obtain nutrients from the challenging soil environment. Two metabolic pathways that use alternative carbon sources to produce acetyl-CoA were highly expressed in soil: the degradation of phenylacetic acid (PAA) and the β-oxidation of fatty acids (Fig 5). The PAA pathway is conserved in 16% of bacteria, which highlights its relevance [54]. In addition, in *R*. *solanacearum*, like in the vast majority of bacteria, the *paaABCDE* genes are clustered in an operon [54]. Moreover, this pathway has been observed to be used by different soil bacteria such as *Pseudomonas putida* to degrade aromatic compounds like lignin [55,56] and use them as a carbon source [57]. The increase in the acetyl-CoA pool produced by fatty acid oxidation and PAA degradation is likely fuelled to the glyoxylate cycle [58] as the genes encoding its two key enzymes (isocitrate lyase and acyl-CoA dehydrogenase) were also highly upregulated in soil (Fig 5). This variation of the tricarboxylic acid cycle enables the utilization of acetate as carbon source under glucose deprivation [59]. The final product, oxaloacetate is likely redirected into gluconeogenesis in soil, as the glycogen and trehalose biosynthetic genes were also upregulated. Both compounds have been described to enhance bacterial survival under osmotic and oxidative stresses likely encountered in this environment [60–62]. Other pivotal genes involved in glyoxylate metabolism, such as the glyoxylate carboligase and glycerate kinase were also strongly induced, highlighting the key role of this compound in soil conditions. Future studies will shed light on the similarities and differences of these enzymes with other soil-bacteria, as well as their relevance for *R*. *solanacearum* soil survival.

In line with the described metabolic rearrangement, a switch in nutrient transport systems was detected, with downregulation of permease xylose, ribose and trehalose transporter genes and upregulation of genes encoding alternative carbon and high affinity transporters. We conclude that *R*. *solanacearum* is a soil bacterium able to degrade aromatic compounds frequent in this environment and that it shows a remarkable metabolic plasticity required to survive and adapt to this habitat.

The observed switch in bacterial metabolism was associated with changes in transcriptional regulators. For example, the two *R*. *solanacearum* genes encoding the global regulator H-NS were upregulated in soil (one being among the top upregulated genes). H-NS modulates expression of genes for adaptation to environmental challenges [63,64] and could thus play a pivotal role controlling the soil transcriptional reprogramming. H-NS also silences DNA acquired through horizontal gene transfer, such as pathogenicity islands [65–67]. In harmony with this, expression of most genes associated with host virulence was repressed in soil (Table 1 and S4 Fig), as observed in other soil-borne pathogens [68] and *R*. *solanacearum* exposed to plant debris present in soil [69]. Since *R*. *solanacearum* is naturally competent, the induction of H-NS expression may act as a sentinel mechanism preventing uncontrolled expression of newly acquired DNA foreign DNA from the soil microbiome.

Remarkably, up-regulation of genes associated with nitrogen metabolism was also characteristic of the soil habitat (S5 Fig). Nitrate respiration, assimilation and the detoxification of intermediate N compounds, has been extensively studied in the xylem, where it is essential for the life and virulence of *R*. *solanacearum* [28,70,71]. Upregulation of all the Nitrogen metabolism genes seems to indicate that the bacterium is carrying out denitrification in the soil. This observation highlights the potential importance of nitrogen metabolism for *R*. *solanacearum* survival in soil and calls attention to the impact of field fertilisation, which could favour *R*. *solanacearum* survival. Induction of denitrification genes could also be due to the microaerophilic environment associated with the natural soil used for all experiments, which is rich in clay. Consistent with limitations in oxygen availability we observed differential expression of the three *R*. *solanacearum* terminal oxidases for aerobic respiration: cytochrome *c aa*_*3*_-type oxidase (*cyo* genes), cytochrome *c cbb*_*3*_-type oxidase (*cco* genes) and cytochrome *bd* oxidase (*cyd* genes) and [70]. *cyo* genes were strongly downregulated in soil whereas *cco* were highly expressed. The *bd* oxidase, which was also upregulated in soil, is prevalent among pathogenic bacteria and plays a role in resistance to small molecules [72].

The soil habitat is a hostile environment where heavy metals, antimicrobials, oxidative stress and temperature and humidity fluctuations affect bacterial growth [73–76]. In line with this, most genes up regulated in soil were associated with coping with an oxidative stress and metal homeostasis. Adaptation of the bacterium to oxidative stress is illustrated by the induction of the redox-sensing transcriptional regulator *oxyR*, which controls expression of many oxidative stress genes [44,77]. The *katE* catalase and *katG* catalase/peroxidase genes are induced by OxyR and were among the most highly upregulated genes in soil (Fig 6A). While deletion of *oxyR* in *R*. *solanacearum* resulted in a clear penalty in virulence [44], deletion of other oxidative stress genes such as *katE* and *dps* did not affect virulence or only marginally [42,78], suggesting that they play a role at other stages during the bacterial life cycle. Remarkably, mutation of *oxyR* and *katG* -but not *katE-* caused a decrease in culturability in soil microcosms (Fig 6C and 6D). Live staining indicated that most bacterial cells remained alive and were likely in the VBNC state. Catalases have been shown to delay the entrance of the soil-borne phytopathogen *Erwinia amylovora* in the VBNC state [79] and to rescue *R*. *solanacearum* from this condition [46]. *R*. *solanacearum* cells that entered the VBNC state have been shown to be able to resuscitate and remain pathogenic [13]. We thus hypothesize that the VBNC cells observed in this work are still infectious. However, given that only a small proportion of cells enter the VBNC state and only a few founder cells are sufficient to cause disease, it is difficult to determine if the VBNC cells are still pathogenic.

Our results demonstrate that *oxyR* and *katG* make a significant contribution to bacterial adaptation to the soil habitat and highlights the need for future research to further characterize the mechanisms associated with the VBNC state in soil.

## Methods

### Bacterial strains, plant growth conditions and inoculations

All strains, plasmids and primers used in this work can be found in S6 Table. The *R*. *solanacearum* strain UY031 (phylotype IIB) isolated from potato tubers in Uruguay [80] carrying the reporter *lux* operon under control of the constitutive *psbA* promoter was used for the transcriptome analysis [29]. For all the other experiments, *R*. *solanacearum* GMI1000 (phylotype I) carrying the reporter *lux* operon under the control of the *psbA* promoter was used. All the *R*. *solanacearum* and *R*. *pseudolanacearum* strains were routinely grown at 28 °C in rich B medium [29] and *E*. *coli* strains in Luria-Bertani (LB) medium at 37 °C. In all cases, the medium was supplemented with ampicillin (50 mg/L), kanamycin (50 mg/L), gentamicin (10 mg/L), or tetracycline (10 mg/L) when needed.

For *in planta* assays, tomato plants (*Solanum lycopersicum* cv. Marmande) were routinely grown in a 30:1:1 mix of Substrate 2 (Klasmann-Deilmann GmbH), perlite and vermiculite for four weeks at 22 °C and 60% relative humidity (RH) under long-day photoperiod conditions (16 h light and 8 h darkness). Before bacterial inoculation, tomato plants were pre-acclimated for three days at infection conditions (27 °C, 12/12 h photoperiod and 60% RH).

Plant inoculations were performed by soil drenching at 10^8^ CFU/mL as described [81]. To measure xylem pH, two 3 cm stem samples were collected per plant 0.5 cm above the soil (bottom) and the 4^th^ internode (top), pH was measured using a pH-indicator strips pH 2.0–9.0 (Merck). The sap was serially diluted and plated in rich B medium with appropriate antibiotics to count bacterial colonies.

### RNA-seq and RT-PCR sample preparation

For the soil samples, a mix of natural soil and sand in a 2:1 ratio (S2 Table) was autoclaved three times (~3 h) and 200 g of soil was inoculated to a final concentration ~10^7^ CFU/g soil and incubated for 3 days at plant infection conditions. For the water samples, bacteria were recovered 48 h after growth in rich B medium plates, washed and resuspended in sterile mineral water (Water A—S1 Table) to a final concentration of ~10^7^ CFU/mL in 250 mL. The suspension was incubated at 28 °C, 100 rpm for 6 hours, centrifuged at 4 °C, and the pellet frozen in liquid nitrogen. As previously done for *in planta* bacterial samples [28], water and soil samples were adjusted to comparable cell densities to avoid a major impact on the transcriptome due to quorum sensing responses, which would mask the detection of environment-specific gene expression changes. For the RT-PCR samples, *R*. *solanacearum* was inoculated in sterile mineral water to a final concentration of ~10^7^ CFU/mL in 250 mL and incubated for 3 days at 28°C, 100 rpm for 3 days. For RNA extraction, bacterial cells were pelleted at 4 °C, and the pellet frozen in liquid nitrogen.

Total RNA was extracted using the SV Total RNA Isolation System kit (Promega) for water samples and the RNA PowerSoil Total RNA Isolation Kit (MO BIO) followed with a rigorous DNAse treatment with the TURBO DNA-free kit (Life Technologies) for soil samples. Only RNA with RIN greater than 7.0 was used for library construction and samples were subjected to bacterial rRNA depletion prior to sequencing on a HiSeq2000 Illumina System apparatus. Reads for the reference rich B medium and the three *in planta* (Potato—*Solanum tuberosum* cv. Desirée) conditions (Apoplast, Early xylem and Late xylem) conditions were recovered from our previously published data [28].

### Real time quantitative PCR (RT-PCR)

Reverse transcription of RNA was performed with the RevertAid first strand cDNA synthesis kit (ThermoScientific) following manufacturer’s instructions. The Sybr Green Master Mix (Sigma Aldrich) was used for RT-PCR with the LightCycler 480 Instrument (Roche Life Science) using primers listed in S6 Table. The phosphoserine aminotransferase gene (serC) was used as a reference gene for normalisation of expression [29].

### Read alignment, mapping and analyses

All the transcriptomic datasets used in this study, were analysed following the same bioinformatic pipeline as summarized in S1B Fig. For the gene enrichment analysis, the UY031 genes were searched for associated GO terms using the OmicsBox software (v. 2.2.4, 82] and the enricher function of the ClusterProfiler package (v. 4.2.2, 83]. For Kyoto Encyclopaedia of Genes and Genomes (KEGG) pathways, terms were downloaded from KEGG API (December 12, 2022, 84]. Genes were also functionally categorised using the EggNOG-mapper [85] to retrieve the Cluster of Orthologous Groups (COG). COG categorisation was curated with information from the KEGG and Uniprot databases [84,86], together with previously published data. This curated classification was used to conduct a hypergeometric test to detect enriched categories among DEGs (v. 4.1.0).

### Mutant construction

Knockout deletion plasmids of the *katG* tandem genes (*RSc0775*/*Rsc0776*) and *oxyR* (*RSc2690)* in *R*. *solanacearum* GMI1000 strain were generated by double-joint PCR as previously described in [42]. The Knockout constructs were linearised and double recombination events selected after natural transformation into *R*. *solanacearum* [87].

For complementation, the tandem *RSc0775/Rsc0776* genes with their promoter were cloned in the *Kpn*I and *Xba*I sites of pRCT-GWY [88], creating pRCT-*PkatG*:*KatG* and *oxyR* was cloned through Gateway reactions (Thermo Fisher) in pRCT-PpsbA-GWY [88], generating pRCT-*PpsbA*:*OxyR*. *R*. *solanacearum* were complemented after integration of the constructs in a permissive site as described [87]. All oligonucleotides used are listed in S6 Table.

The *PrhJ* promoter was cloned the in *Avr*II and *Kpn*I sites of pRCG-GWY [29] and the entire *luxCDABE* operon from pRCGent-*Pep*:*lux* [29] was cloned on the *Kpn*I and *Sfi*I sites to generate pRCG-*PprhJ*:*lux*. All constructs were *Sfi*I-digested and genome integrated by natural transformation. All transformants were confirmed by PCR amplification.

### Reporter expression

Overnight cultures of the reporter strains were washed and inoculated in water (native pH or adjusted pH) supplemented or not with rich B medium (1/50, 1/10 or 1/5 final concentrations) to a final concentration of 10^8^ CFU/mL. A list of all the mineral waters used in this work can be find in S1 Table. Bacterial suspensions were kept in water at 28 °C with shaking at 180 rpm and aliquots collected over time in triplicate experiments to measure luminescence and absorbance [89]. Luminescence results were expressed as relative light units (RLU) values divided by 1000 and normalized by the bacterial density (OD_600_).

### Soil agar and microcosms experiments

Soil extract agar was prepared by mixing 400 g of natural soil (S2 Table) in 1 L of water. The mix was autoclaved, kept at room temperature for 24 h and centrifuged 10 min at 5000 rpm. The liquid phase was recovered, 15 g/L of agar added, and pH adjusted to 6.8–7[90]. Washed *R*. *solanacearum* overnight cultures adjusted to 10^8^ CFU/mL were serially diluted and plated on soil extract agar plates supplemented with appropriate antibiotics. After 48 h at 28 °C, bacterial colonies were counted.

For microcosm assays, 15 g of autoclaved soil were inoculated with 3.5 mL of bacteria (corresponding to the soil field capacity) to reach a final concentration of ~3 × 10^8^ CFU/g soil [46]. Flasks were incubated under regular infection conditions and bacterial culturability measured by collecting ~0.5 g of soil at different times, homogenising them in 1 mL of water and plating serial dilutions onto modified antibiotic-containing SMSA medium [91]. Colonies were counted after 48 h incubation at 28 °C and normalised by the soil weight.

### Viable cell staining

Cells were stained using a BacLight LIVE/DEAD bacterial viability kit (ThermoFisher), details are listed in S8 Fig.

## Supporting information

S1 Dataset DEGs in the two environmental conditions (Soil and Water) and in the three *in planta* conditions (Apoplast, Early xyem and Late xylem) compared to the reference rich B medium (phi). Differentially expressed genes were selected with DESeq2 (p-adj > 0.01, log2 FC ± 1.5).
(XLSX)

S1 Table List of waters used in this work. Native pH and location (also illustrated in a map) is indicated for each water.
(XLSX)

S2 Table Chemical analysis of the natural soil used in the study. (XLSX)

S3 Table List of genes correspoding to the intersections shown in the UpsetR plots (Fig 1B and 1D). (XLSX)

S4 Table TAG enrichment analysis summary. Percentage of DEGs in each of the manually curated functional categories and the hypergeometric enrichment text are shown for the environmental soil and water conditions for the up- (_UP) and downregulated (_DOWN) genes. Hypergeometric statistical analysis was conducted in R package stats.
(XLSX)

S5 Table Output tables of the KEGG and GO enrichment analysis conducted on general and exclusive up- and downregulated DEGs from water and soil conditions. (XLSX)

S6 Table Bacterial strains, plasmids, and oligonucleotides used in this work. (XLSX)

S1 Fig Experimental set-up and differentially expressed genes (DEGs). **A**) RNA sampling conditions from environmental (soil and mineral water) and previously obtained samples (rich B medium reference and three *in planta* conditions). **B**) Transcriptomic data analysis pipeline. First, raw RNA-seq data quality was evaluated with FastQC (v.0.11.5), trimmed with trimGalore (v.0.6.1) and potential rRNA contaminants were filtered out with the SortMeRNA software (v.4.2.0). Reads were mapped with Bowtie2 (v. 2.4.4) and alignments quantified with FADU v. 1.8. The *R*. *solanacearum* UY031 genome GCF_001299555.1_ASM129955v1 was used. DEG analyses were performed with Deseq2 (v. 1.34.0). Genes with |log2(fold-change)|>1.5 and adjusted p-value <0.01 were considered as differentially expressed (DEG) when compared to the reference medium. The UpsetR package (v. 1.4.0) (90) was used to detect unique DEG and intersections among the in the different conditions. Deseq2 transformed counts normalized for sample size were used for principal component analysis. **C**) DEGs in the different conditions. Bars show the total number of up- (yellow) and downregulated (blue) genes for each condition compared to the reference Rich B medium. The line graph and the right Y axis indicate the percentage of DEGs. Drawings created with BioRender.
(TIFF)

S2 Fig GO and KEGG enrichment analyses of the environmental conditions. Dot plots of the KEGG (left) and GO (right) enrichment analyses of differentially expressed genes (DEGs) from soil (brown) and water (blue) conditions. Dot sizes represent the number of genes associated with each term and dot colour indicates the p. adjusted value. The gene ratio represented in the X axis is the proportion of associated genes to a term from the total gene set. The DEGs were extracted with DEseq2 using the thresholds: p-adj.value > 0.01 and log 2 FC ± 1.5 and ClusterProfiler was used to calculate the enrichment.
(TIF)

S3 Fig Time-course expression of the *PhrpB*::*Lux* reporter in strains disrupted for the different T3SS regulatory genes after resuspension in water. *R*. *solanacearum* cultures grown overnight in rich B medium were washed and diluted to OD_600_ = 0.1 in water and luminescence and OD_600_ values were measured over a 24h period. Relative luminescence units (RLU) were normalised by bacterial concentration measured as OD_600_. RLU values were divided by 1000 to facilitate visualisation.
(TIFF)

S4 Fig Induction of the type 3 secretion system (T3SS) by basic pH in all natural water sources tested. Time-course expression of *prhG*, *hrpB* and *hrpY* reporter strains at native basic (A to F) or neutral pH (G) and after pH neutralisation with HCl or alkalinisation with KOH. For each time point, luminescence was measured (RLU) and normalised by OD_600_. All values were divided by 1000 to facilitate visualisation. Letters indicate the water sources detailed in the methods section.
(TIFF)

S5 Fig Induction of nitrogen metabolism genes in soil. **A**) Representation of the main components of the nitrogen metabolism and their expression in different conditions (log2 fold change with respect to rich B medium). Genes depicted in A correspond to genes highlighted in bold in B. **B**) Heatmap representation of the log2 fold change in expression with respect to growth in rich B medium for all the genes classified in the nitrogen metabolism group. The colour palette ranges from blue (downregulated) to yellow (upregulated genes) as indicated in the key. Locus names are presented without the preceding letters RSUY_.
(TIFF)

S6 Fig Expression of all stress response and the type 3 secretion system (T3SS) and type 3 effector gene groups. Heatmap representation of gene log2 fold change with respect to the rich B medium in the different conditions for **A**) All stress response genes and **B**) T3SS and T3E gene categories according to the curated classification (see M&Ms). The colour palette ranges from blue (downregulated) to yellow (upregulated genes) as indicated in the key. Locus names are presented without the preceding letters RSUY_.
(TIFF)

S7 Fig Expression of key genes associated with oxidative stress in soil and water at 3 dpi. Real time q-PCR was performed to validate gene expression in soil and water at 3 dpi. The genes selected were *ahpC* (RSUY_35830), *katE* (RSUY_45660), *katG* (RSUY_09930) and *oxyR* (RSUY_07740). Relative expression of target genes was calculated with the DeltaCt method using the geometric mean of the housekeeping gene *serC* (RSUY_11010) as reference. Error bars represent standard error across three technical replicates.
(TIFF)

S8 Fig Viability of *R*. *solanacearum* cells in soil microcosms. **A**) Representative confocal laser scanning microscope images of bacterial cells extracted from soil microcosms at 28 dpi. Cells were stained using the BacLight LIVE/DEAD bacterial viability kit. Green cells represent viable cells and red represents dead cells. Scale bar represents 50 μm. **B**) Quantification of dead cells at different time points of soil microcosms based in BacLight LIVE/DEAD staining. Cells were quantified using Fiji ImageJ. Detailed methods:0.5 g from soil microcosms experiments was resuspended with 1 mL MilliQ water, samples were vortexed and soil particles allowed to sediment. Supernatants were recovered, centrifuged at 1000 rpm for 5 min to eliminate soil and supernatant was transferred to a fresh tube and cells were pelleted by centrifugation at 10000 rpm, 5 min and finally, cells were resuspended in 300 μL 0.85% NaCl. Then, 50 μL of cells were stained with 0.2 μL of BacLight LIVE/DEAD bacterial viability kit (ThermoFisher), incubated for 5 min in the dark and visualized promptly with a confocal microscope. Green SYTO9 fluorescence was measured with excitation at 488 nm and emission at 500–520 nm and red PI fluorescence was measured by excitation at 561 nm and emission at 610–630 nm. The alive control was prepared by washing an overnight culture with 0.85% NaCl and the dead control was prepared by killing cells from the overnight culture with 70% isopropanol for 1 h, followed by centrifugation of cells and resuspension in 0.85% NaCl. The number of alive and dead cells was quantified using ImageJ by setting a “threshold method = Moments” and particles size = 0.5-Infinity.
(TIFF)
