# Supplementary figures and images for: Gene expression changes throughout the life cycle allow a bacterial plant pathogen to persist in diverse environmental habitats
Source: PLoS Pathog. 2023 Dec 19;19(12):e1011888. doi: 10.1371/journal.ppat.1011888 (PMC10763947; doi:10.1371/journal.ppat.1011888)

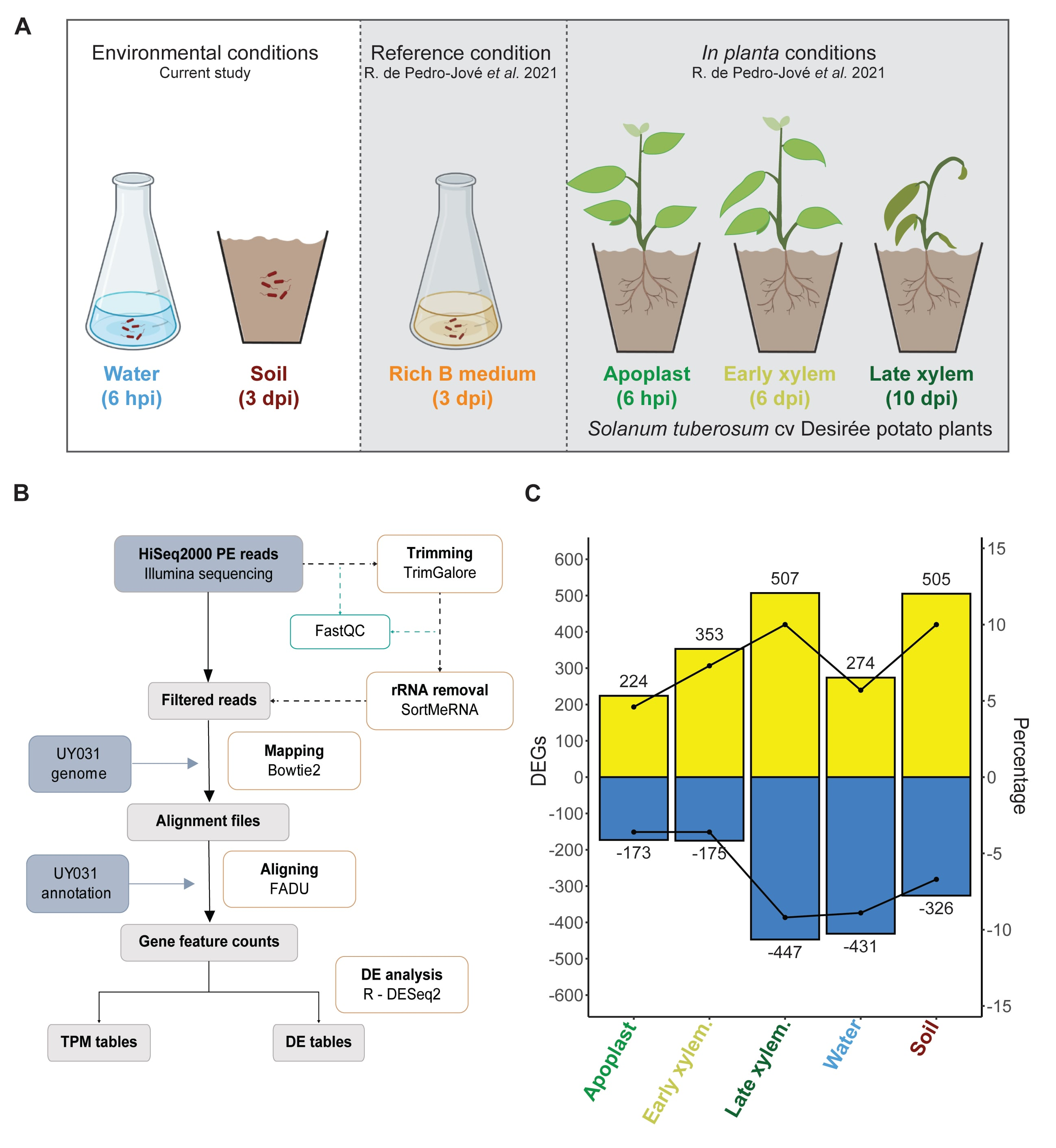

Supplement: S1 Fig — A) RNA sampling conditions from environmental (soil and mineral water) and previously obtained samples (rich B medium reference and three in planta conditions). B) Transcriptomic data analysis pipeline. First, raw RNA-seq data quality was evaluated with FastQC (v.0.11.5), trimmed with trimGalore (v.0.6.1) and potential rRNA contaminants were filtered out with the SortMeRNA software (v.4.2.0). Reads were mapped with Bowtie2 (v. 2.4.4) and alignments quantified with FADU v. 1.8. The R. solanacearum UY031 genome GCF_001299555.1_ASM129955v1 was used. DEG analyses were performed with Deseq2 (v. 1.34.0). Genes with |log2(fold-change)|>1.5 and adjusted p-value <0.01 were considered as differentially expressed (DEG) when compared to the reference medium. The UpsetR package (v. 1.4.0) (90) was used to detect unique DEG and intersections among the in the different conditions. Deseq2 transformed counts normalized for sample size were used for principal component analysis. C) DEGs in the different conditions. Bars show the total number of up- (yellow) and downregulated (blue) genes for each condition compared to the reference Rich B medium. The line graph and the right Y axis indicate the percentage of DEGs. Drawings created with BioRender. (TIFF) [file ppat.1011888.s008.tiff]

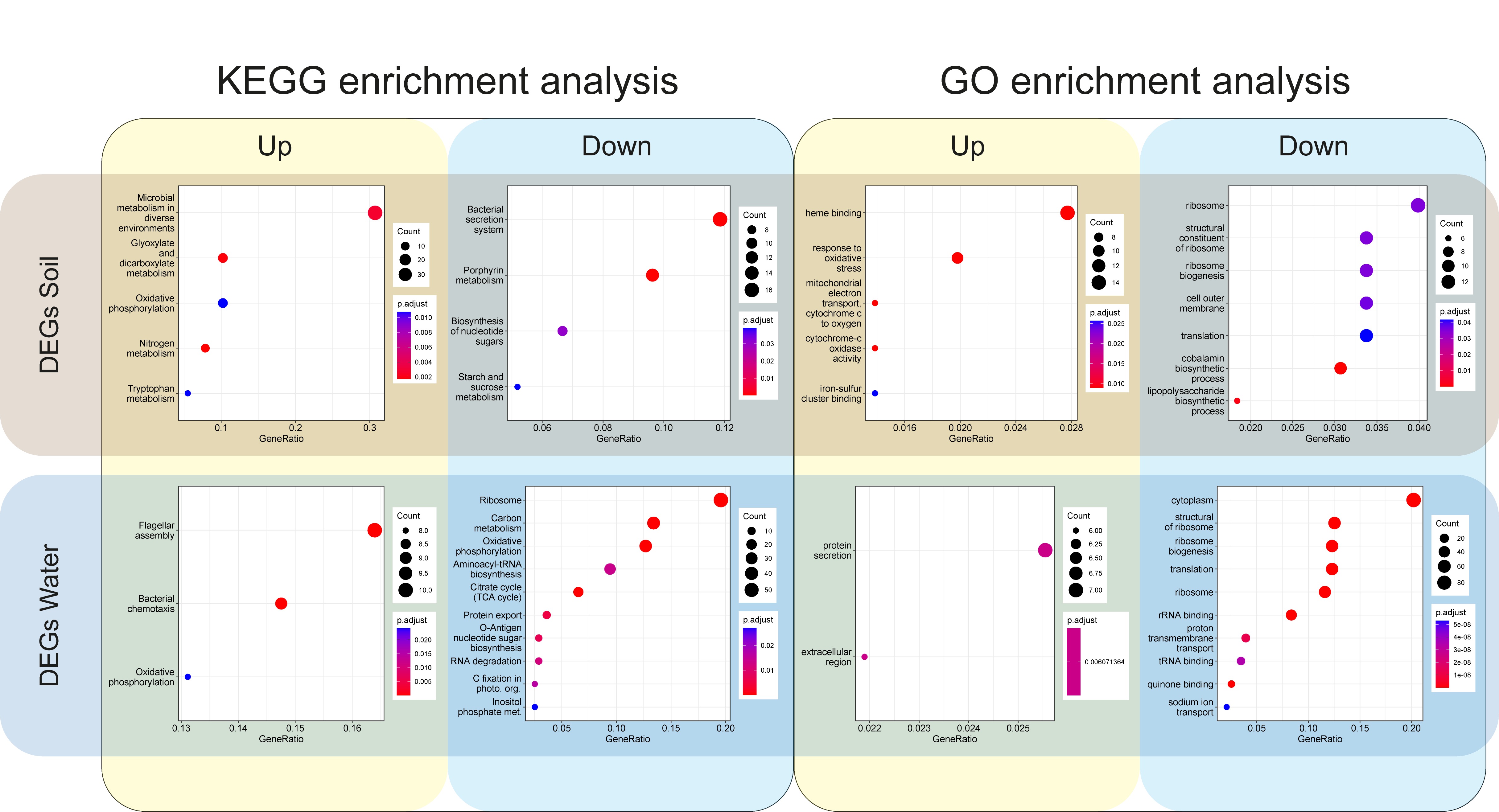

Supplement: S2 Fig — Dot plots of the KEGG (left) and GO (right) enrichment analyses of differentially expressed genes (DEGs) from soil (brown) and water (blue) conditions. Dot sizes represent the number of genes associated with each term and dot colour indicates the p. adjusted value. The gene ratio represented in the X axis is the proportion of associated genes to a term from the total gene set. The DEGs were extracted with DEseq2 using the thresholds: p-adj.value > 0.01 and log 2 FC ± 1.5 and ClusterProfiler was used to calculate the enrichment. (TIF) [file ppat.1011888.s009.tif]

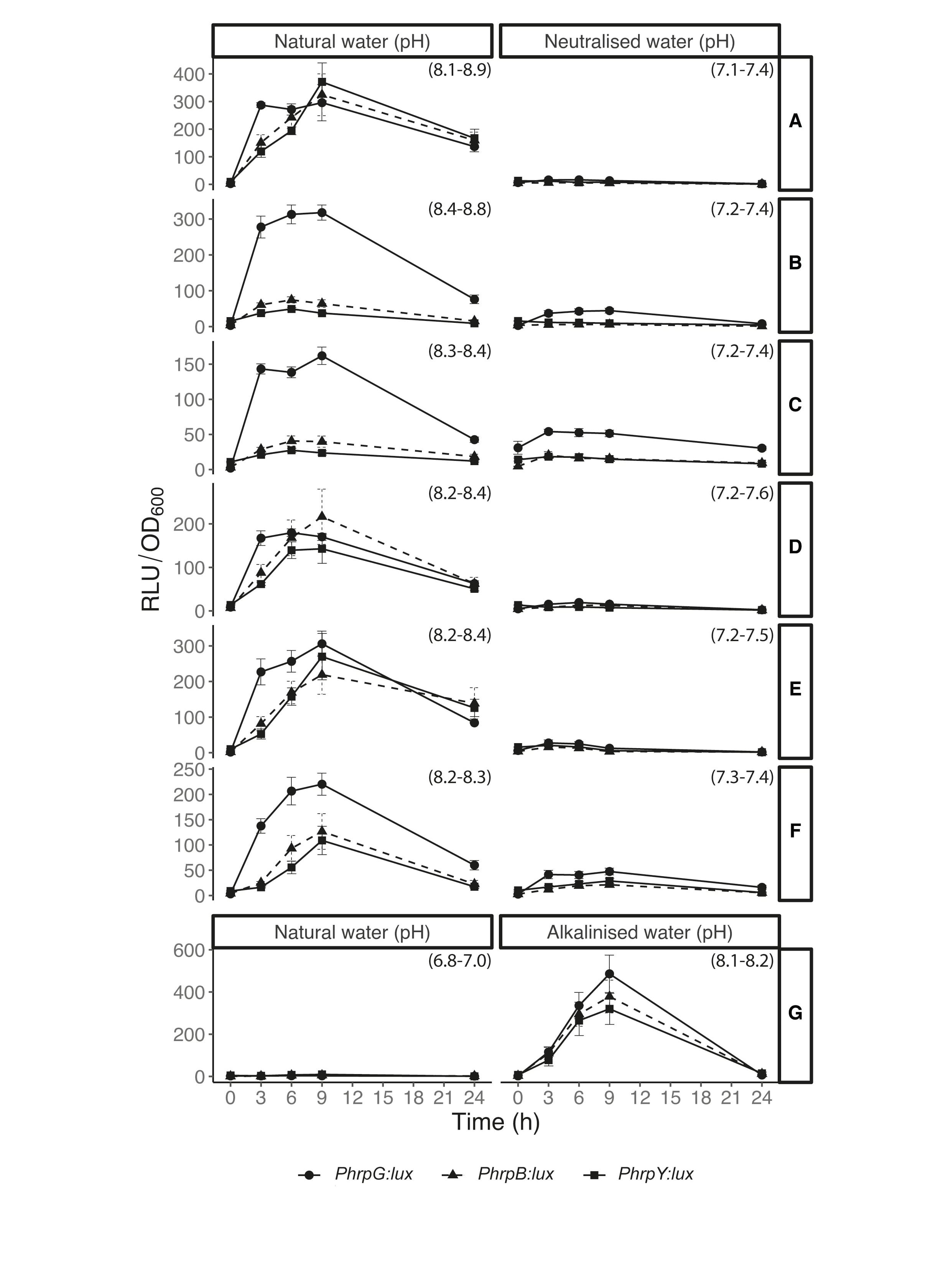

Supplement: S4 Fig — Time-course expression of prhG, hrpB and hrpY reporter strains at native basic (A to F) or neutral pH (G) and after pH neutralisation with HCl or alkalinisation with KOH. For each time point, luminescence was measured (RLU) and normalised by OD600. All values were divided by 1000 to facilitate visualisation. Letters indicate the water sources detailed in the methods section. (TIFF) [file ppat.1011888.s011.tiff]

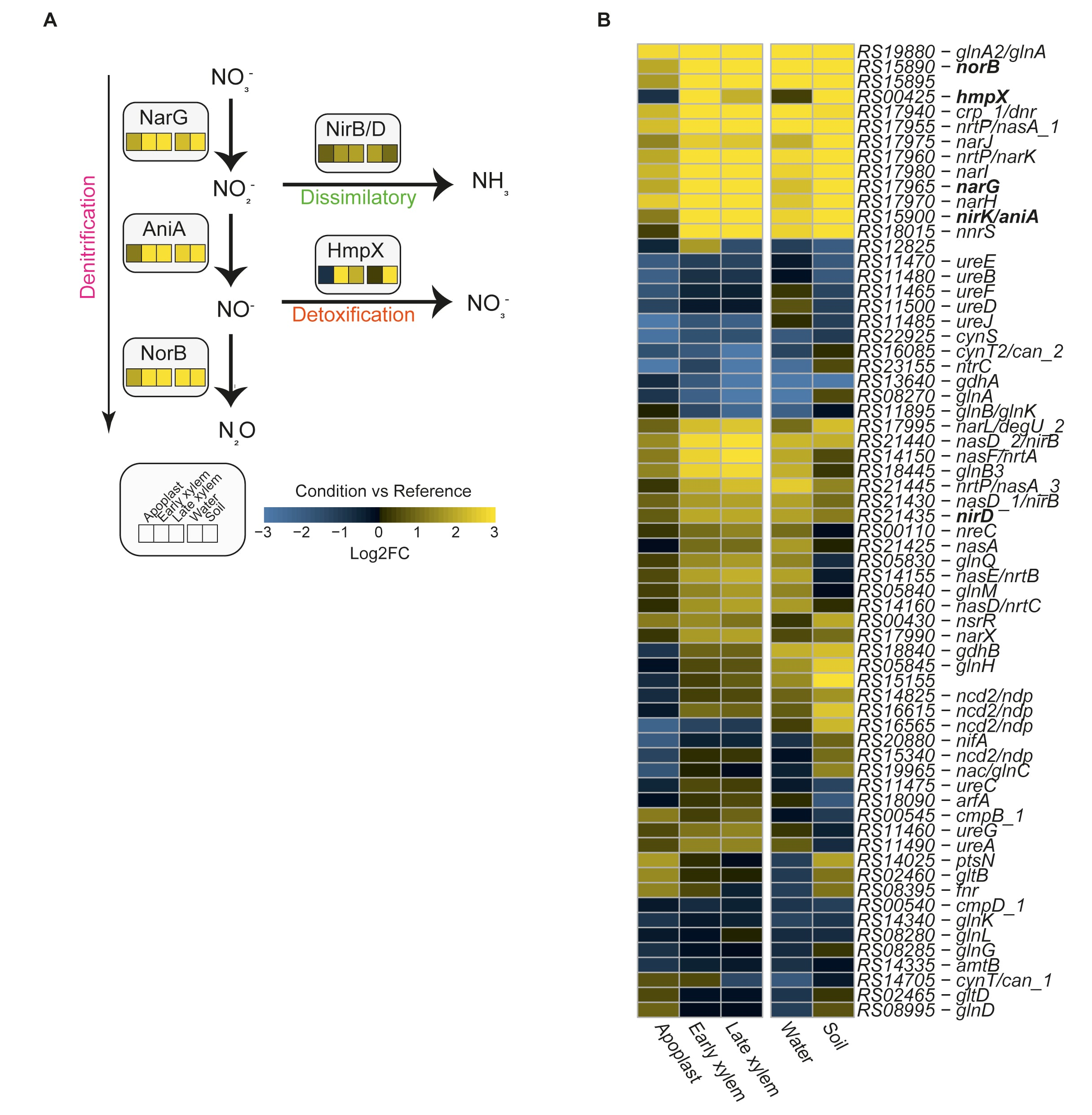

Supplement: S5 Fig — A) Representation of the main components of the nitrogen metabolism and their expression in different conditions (log2 fold change with respect to rich B medium). Genes depicted in A correspond to genes highlighted in bold in B. B) Heatmap representation of the log2 fold change in expression with respect to growth in rich B medium for all the genes classified in the nitrogen metabolism group. The colour palette ranges from blue (downregulated) to yellow (upregulated genes) as indicated in the key. Locus names are presented without the preceding letters RSUY_. (TIFF) [file ppat.1011888.s012.tiff]

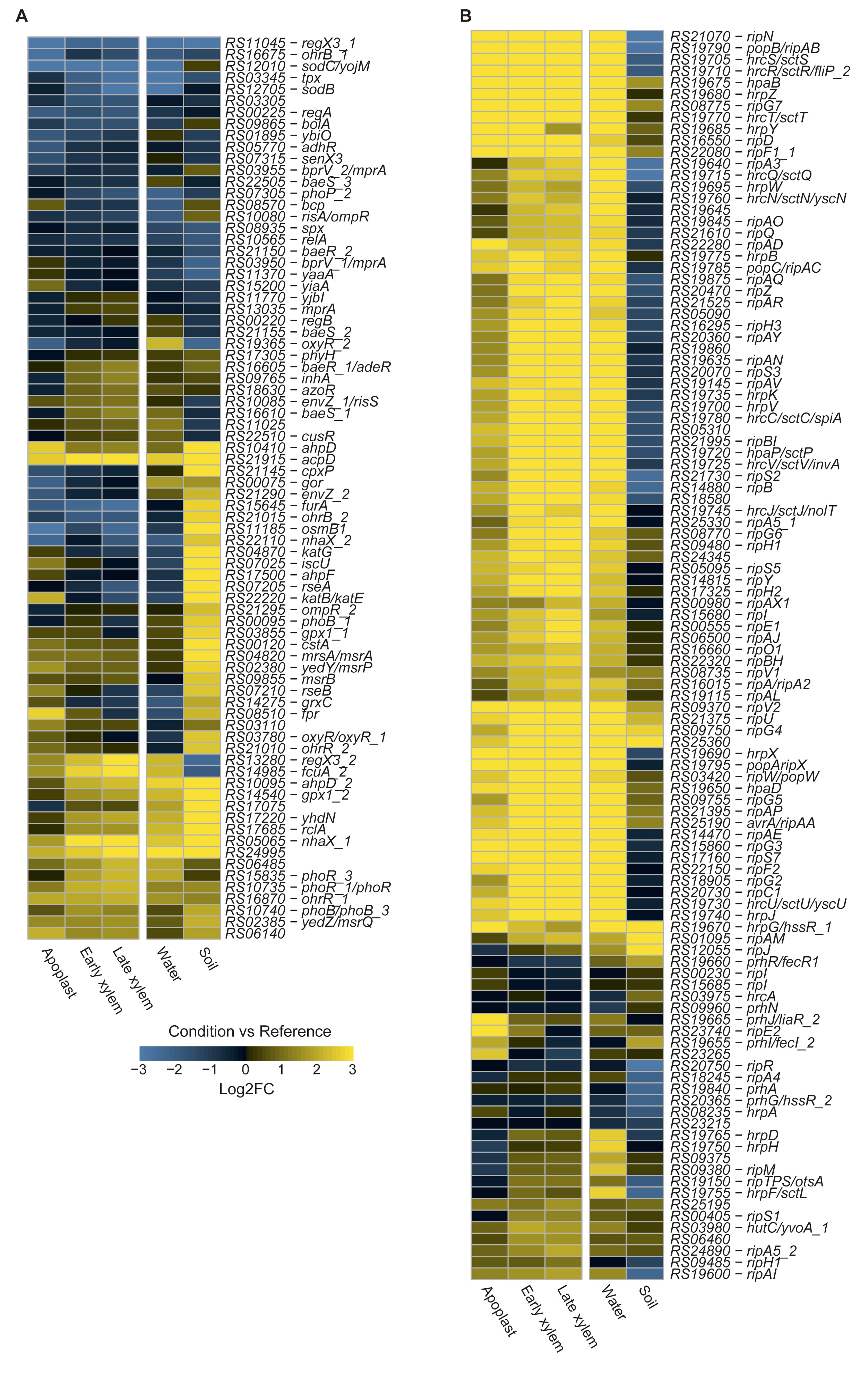

Supplement: S6 Fig — Heatmap representation of gene log2 fold change with respect to the rich B medium in the different conditions for A) All stress response genes and B) T3SS and T3E gene categories according to the curated classification (see M&Ms). The colour palette ranges from blue (downregulated) to yellow (upregulated genes) as indicated in the key. Locus names are presented without the preceding letters RSUY_. (TIFF) [file ppat.1011888.s013.tiff]
